# Catalpol promotes the generation of cerebral organoids with oRGs through activation of STAT3 signaling

**DOI:** 10.1002/btm2.10746

**Published:** 2024-12-29

**Authors:** Yoo‐Jung Lee, Byounggook Cho, Daeyeol Kwon, Yunkyung Kim, Saemin An, Soi Kang, Jongpil Kim

**Affiliations:** ^1^ Department of Chemistry Dongguk University Seoul Republic of Korea

**Keywords:** Catalpol, cerebral organoids, outer radial glia cells, STAT3 signaling

## Abstract

The generation of human cortical organoids containing outer radial glia (oRG) cells is crucial for modeling neocortical development. Here we show that Catalpol, an iridoid glucoside derived from *Rehmannia glutinosa*, significantly enhances the generation of cerebral organoids with expanded oRG populations and increased neurogenic potential. Catalpol‐treated organoids exhibited thicker ventricular zone/subventricular zone (VZ/SVZ) and outer subventricular zone (oSVZ) regions, with increased numbers of SOX2 + HOPX+ and SOX2 + TNC+ oRG cells and elevated expression of oRG markers HOPX and FAM107A. We found that Catalpol promoted oRG generation through non‐vertical divisions of ventricular radial glia (vRG) cells, indicating enhanced oRG generation via asymmetrical divisions. Furthermore, we demonstrated that Catalpol augmented oRG cell numbers through activation of the STAT3 signaling pathway. These findings highlight Catalpol's potential in promoting the generation of cerebral organoids with expanded oRG populations and increased neurogenic potential through STAT3 activation, offering new insights into neocortical development modeling.

**Translational Impact Statements**

The generation of cerebral organoids containing expanded populations of outer radial glia (oRG) cells represents a critical advancement in modeling human neocortical development and understanding neurological disorders. This study identifies Catalpol, a naturally derived iridoid glucoside, as a potent enhancer of oRG generation in cerebral organoids. By promoting non‐vertical divisions of ventricular radial glia (vRG) cells and activating the STAT3 signaling pathway, Catalpol significantly increases the neurogenic potential and structural complexity of these organoids, particularly within the outer subventricular zone (oSVZ). These findings not only provide valuable insights into the mechanisms of oRG generation but also offer a novel approach for enhancing organoid‐based models of cortical development. This has profound implications for translational research, including the study of neurodevelopmental disorders, high‐throughput drug screening, and the development of regenerative therapies targeting cortical abnormalities.

## 1 INTRODUCTION

The generation of cerebral organoids from human induced pluripotent stem cells (iPSCs) has emerged as a powerful tool for modeling human brain development and disease.^1^, ^2^ These organoids can recapitulate many aspects of the structural and functional complexity of the human brain, providing valuable insights into neurodevelopmental processes and offering a platform for drug testing and disease modeling.^3^, ^4^ A critical component of these models is the presence of outer radial glia (oRG) cells, which are crucial for the expansion and complexity of the human cortex.^5^, ^6^ Thus, the generation of cerebral organoids with a well‐defined outer subventricular zone (oSVZ) containing oRG cells is essential for accurately mimicking neocortical architecture and function. Recent studies have improved the generation of organoids with well‐defined oSVZ containing oRG cells. Qian et al. developed cortical spheroids with abundant oRG cells, while Bershteyn et al. used oRG‐rich organoids to model Miller‐Dieker syndrome.^7^, ^8^ These studies highlight the importance of oRG cells in generation of human cortical organoids. However, generating cerebral organoids with consistent oRG populations and uniform structural features can be difficult due to the absence of proper differentiation process.^9^, ^10^ Thus, ensuring the proper differentiation and maturation of these organoids with oRGs in the 3D culture environment is crucial for their potential application in disease modeling and regenerative medicine.

Catalpol, an iridoid glucoside extracted from the root of *Rehmannia glutinosa*, has been shown to promote neurogenesis and inhibit apoptosis, and reduce inflammation in various models of the diseases. For examples, enhanced neural differentiation and improved survival of mature neurons in the adult hippocampus in a post‐traumatic stress disorder (PTSD) model or stroke models following Catalpol administration.^11^, ^12^ Moreover, Zheng et al. demonstrated a marked reduction in pro‐inflammatory cytokine levels following catalpol treatment, suggesting its potential as an anti‐inflammatory agent.^13^ Similarly, Huang et al. and Wang et al. elucidated catalpol's neuroprotective effects in Alzheimer's and Parkinson's disease models, mediated through the attenuation of oxidative stress and neuronal apoptosis.^14^, ^15^, ^16^, ^17^ Of particular interest is catalpol's effect on key signaling pathways, notably the STAT3 cascade. Dong et al. identified catalpol as a potent activator of STAT3 signaling, which is especially intriguing given the pathway's established involvement in neurogenesis and neural stem cell differentiation.^18^ Given the significant effects of STAT3 signaling on SVZ generation during brain development, we hypothesized that Catalpol could enhance the generation of cerebral organoids enriched with oRGs through activation of the STAT3 signaling pathway.^19^, ^20^

In this study, we demonstrate that Catalpol treatment leads to the efficient generation of cerebral organoids with well‐defined oSVZ regions enriched with oRG cells. This is achieved through the promotion of non‐vertical divisions of ventricular radial glia (vRG) cells mediated by the activation of STAT3 signaling. Furthermore, Catalpol‐treated organoids exhibited enhanced neurogenic capacity of differentiation into both deeper and upper cortical layers. These findings suggest that Catalpol could serve as a valuable tool for improving the reproducibility and scalability of cerebral organoid production with oRGs, thereby enhancing their utility in neurodevelopmental studies and therapeutic applications. By examining the role of Catalpol in STAT3‐mediated oRG generation, we offer new insights into the molecular pathways that drive human neocortical development and highlight the potential of Catalpol as a tool for enhancing the fidelity of cerebral organoid models.

## 2 RESULTS

### 2.1 Efficient generation of cerebral organoids containing oRG with Catalpol treatment

**FIGURE 1:**
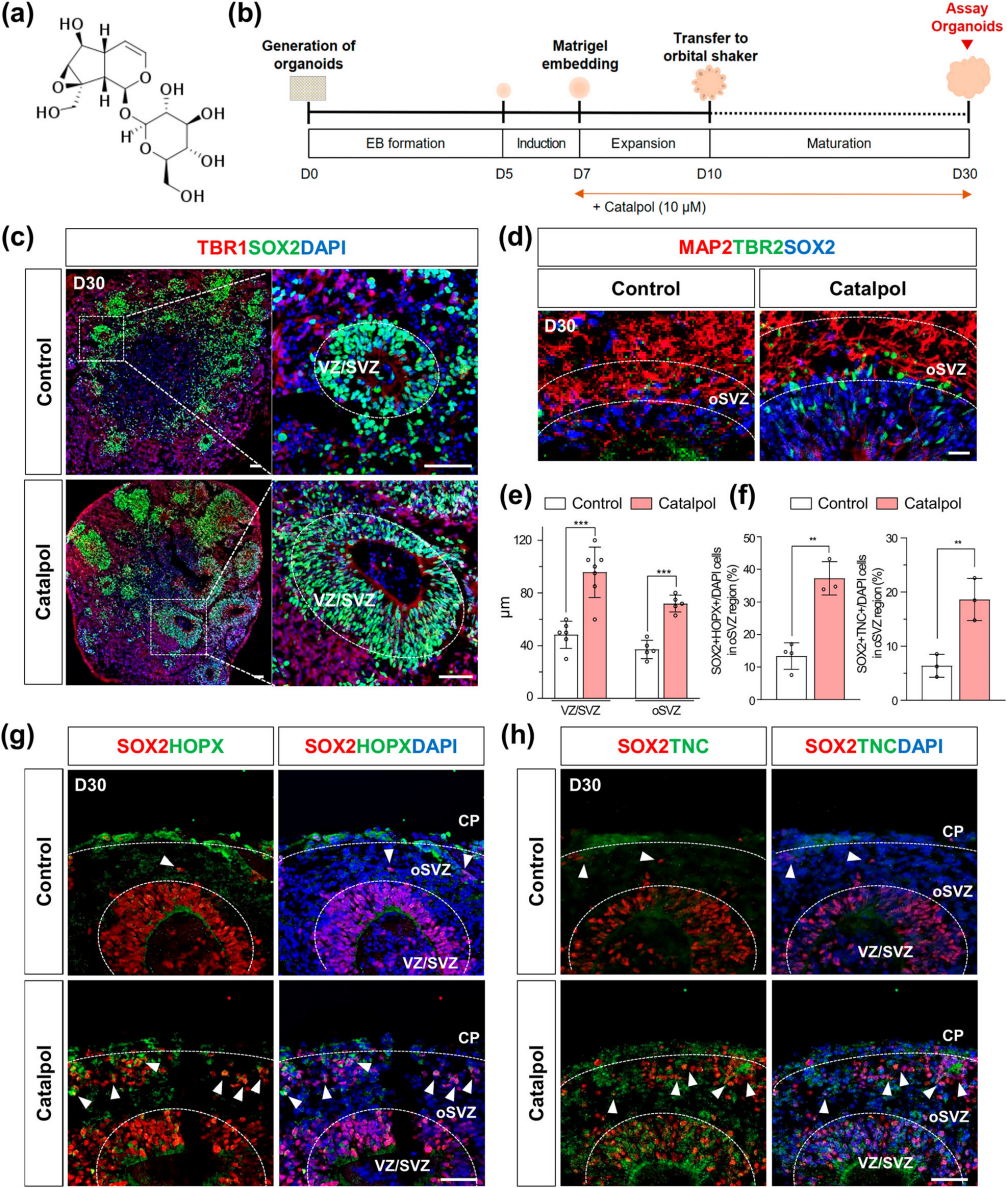
Catalpol increases the development of VZ/SVZ regions and the number of outer radial glia (oRG) in cerebral organoids. (a) Chemical structure of Catalpol. (b) Schematic outline of cerebral organoids differentiation protocol with or without Catalpol (10 μM) treatment. (c) Immunofluorescence staining for TBR1 (red), SOX2 (green), and DAPI (blue) in day 30 control and Catalpol‐treated organoids. Scale bar, 50 μm. (d) Immunofluorescence staining for MAP2 (red), TBR2 (green), and SOX2 (blue) in day 30 control and Catalpol‐treated organoids. Scale bar, 20 μm. (e) Thickness of VZ/SVZ region and oSVZ region in day 30 control and Catalpol‐treated organoids. Data represent mean ± SEM. two‐tailed Student's t‐test, ***p < 0.001; *n* = 5 per group. (f) Quantification of SOX2 + HOPX+ and SOX2 + TNC+ oRG cells in oSVZ region. Data represent mean ± SEM. two‐tailed Student's t‐test, **p < 0.01; *n* = 3 per group (g) Immunofluorescence staining for SOX2 (red), HOPX (green), and DAPI (blue) in day 30 control and Catalpol‐treated organoids. Scale bar, 50 μm. (h) Immunofluorescence staining of SOX2 (red), TNC (green), and DAPI (blue) in day 30 control and Catalpol‐treated organoids. Scale bar, 50 μm. Arrows represent SOX2 + HOPX+ and SOX2 + TNC+ oRG cells. The dotted lines highlight the regions of the VZ/SVZ, oSVZ, and cortical plate (CP). Scale bar, 50 μm.

The in vitro generation of human cerebral organoids with oRG is critical for effectively modeling neocortical development. STAT3 signaling has been found to play a crucial role in the generation of cerebral organoids containing oRG cells. Given that Catalpol, an iridoid glucoside extracted from the root of *Rehmannia glutinosa* Libosch (Figure 1a), is a potent activator of STAT3 signaling,^18^ we examined whether Catalpol‐induced STAT3 activation can promote the generation of human cerebral organoids with a well‐defined oSVZ structure. Cerebral organoids were prepared from human iPSCs, with Catalpol treatment starting at the neural ectoderm induction step on day 7 and continuing until the day of assay on day 30 (Figure 1b).

Next, we performed immunostaining with SOX2 and TBR2 to examine whether Catalpol treatment could promote well‐defined VZ/SVZ and oSVZ regions with distinct populations of oRGs in cerebral organoids. As shown in Figures 1d and S2d, the regions containing Tbr2+ and Sox2− cells were subjected to oSVZ and the regions containing CTIP2+ cells were defined to cortical plate (CP). Interestingly, Catalpol‐treated organoids exhibited markedly distinct VZ/SVZ and oSVZ regions, with significantly greater thickness in these areas compared to those of controls (Figure 1c–e). This observation indicates that Catalpol induces both neural progenitor cells (NPCs) and intermediate progenitor cells (IPCs) in the VZ/SVZ and oSVZ regions, respectively. Moreover, we observed a significant increase in the number of SOX2 + HOPX+ and SOX2 + TNC+ cells, potentially marking oRG cells in the oSVZ region, in Catalpol‐treated organoids (Figure 1f–h). Consistent with previous results in Figure 1g,h, gene expression analysis showed a significant increase in oRG markers such as HOPX and FAM107A, while the expression of vRG and IPC markers showed a slight increase in Catalpol‐treated organoids (Figure S1b). Additionally, we analyzed Ki67+, PAX6+ cells in the VZ/SVZ and Ki67+, HOPX+ cells in the oSVZ regions to assess whether Catalpol treatment enhances proliferation in these distinct compartments (Figure S1c,d). PAX6 and HOPX are specific markers of vRG and oRG in the VZ/SVZ and oSVZ regions, respectively. Our results showed that Catalpol treatment enhances both general progenitor cell proliferation in the VZ/SVZ and oSVZ, but it has a particularly strong increase in Ki67+, HOPX+ cells in the oSVZ, indicating the effect on promoting basal mitosis in the oSVZ and supporting the oRG proliferation and expansion in this region (Figure 1g,h).

Moreover, to assess the induction of forebrain identity, we first examined FOXG1 expression at day 30 in both control and Catalpol‐treated organoids (Figure S1a). We observed show that cerebral organoids exhibited forebrain specification as they matured, with no significant difference in FOXG1 expression between control and Catalpol‐treated organoids. This suggests that Catalpol treatment does not impact the establishment of forebrain identity in these organoids. Furthermore, we observed an increased number of neural stem cells (NSCs) such as qNSCs, aNSCs, and neuroblasts, with no significant increase in astrocytes in the Catalpol‐treated organoids (Figure S2a). This suggests that Catalpol treatment primarily enhances a population of NSCs, rather than promoting increase astrocytes.

Additionally, immunofluorescence analysis revealed elevated levels of the neuronal markers TUJ‐1 and NeuN, which indicate enhanced neuronal differentiation and maturation in Catalpol‐treated organoids (Figure S2b). Consistently, both the expression and number of DCX‐positive cells showed a significant increase, suggesting that Catalpol promotes the proliferation of neuroblasts and contributes to ongoing neurogenesis within the organoids (Figure S2c,e). The cortical layer marker CTIP2, which labels deep‐layer cortical neurons, also exhibited improved layer formation, with more defined and organized structures in Catalpol‐treated organoids (Figure S2d). This organized layer formation highlights the role of Catalpol in supporting the establishment of cortical architecture. Taken together, these findings indicate that Catalpol treatment not only enhances the overall neurogenic environment but also promotes oRG population expansion. This likely contributes to the observed increase in neurogenic markers, improved cortical layer organization, and a subsequent increase in mature neurons within the organoids.

### 2.2 Catalpol promotes the generation of oRG by the asymmetrical division of vRG

**FIGURE 2:**
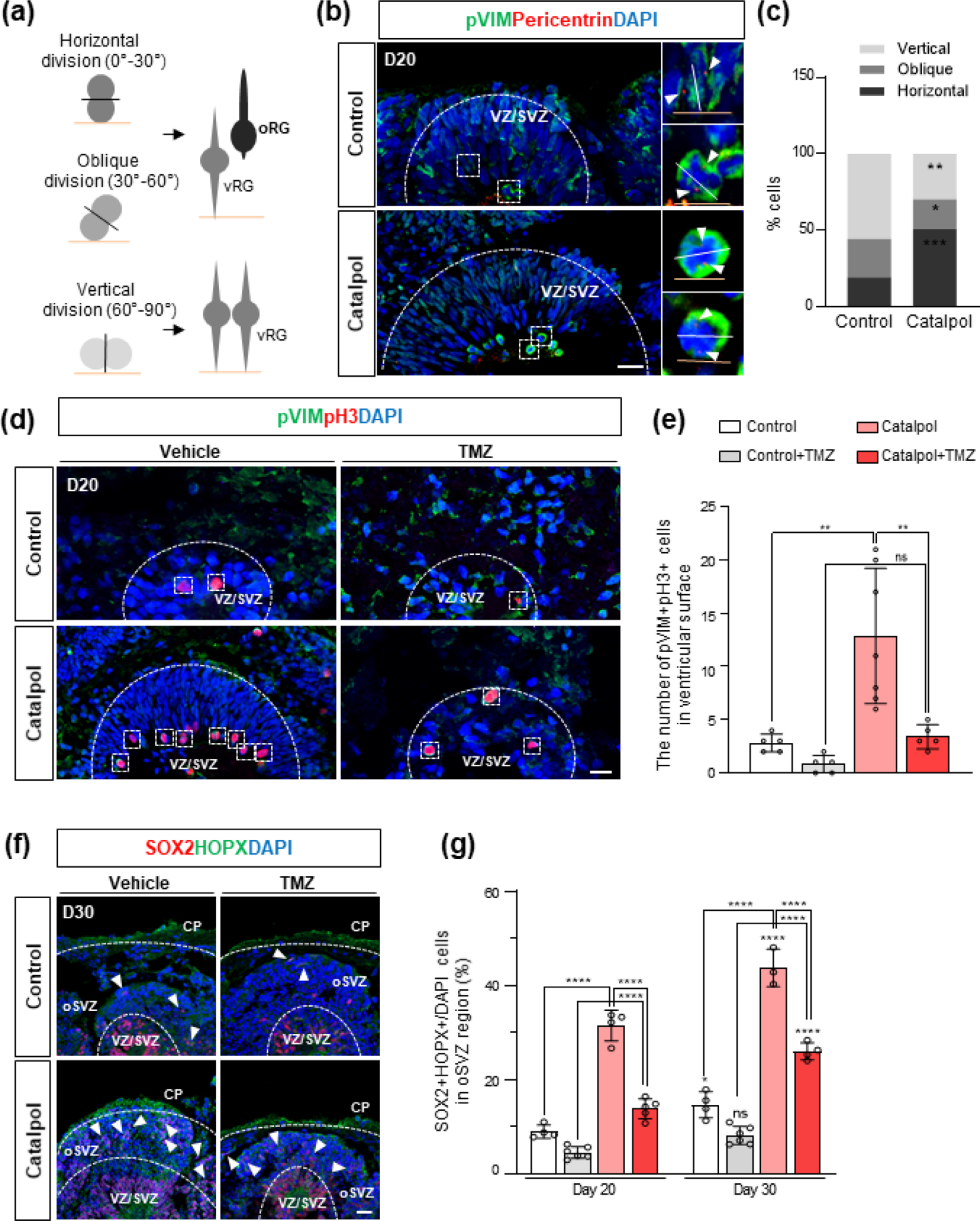
Enhanced generation of outer radial glia (oRG) by non‐vertical division of mitotic ventricular radial glia (vRG) in Catalpol‐treated organoids. (a) Schematic of vRG cells undergoing mitosis at the ventricular surface (solid orange line) with vertical (60–90°), oblique (30–60°) and horizontal (0–30°) cleavage angles. Mitotic spindle orientation was analyzed with respect to the ventricular surface. (b) Immunofluorescence staining for p‐Vimentin (pVIM; green), pericentrin (red), and DAPI (blue) in day 20 control and Catalpol‐treated organoids. The dotted square lines highlight the vRG division. Arrows represent centrosomes. Scale bar, 20 μm. (c) Quantification of mitotic spindle orientation in day 20 control and Catalpol‐treated organoids. Data represent mean. Two‐tailed Student's t‐test, *p < 0.05, *p < 0.01, and ***p < 0.001; *n* = 3 per group. (d) Immunofluorescence staining for pVIM (green), phospho‐histone H3 (pH 3; red), and DAPI (blue) in day 20 control and Catalpol‐treated organoids with or without TMZ. Scale bar, 20 μm. (e) The number of pVIM+pH 3+ cells in ventricular surface. Data represent mean ± SEM. two‐way ANOVA, **p < 0.01; *n* = 3 per group. (f) Immunofluorescence staining for SOX2 (red), HOPX (green), and DAPI (blue) in day 30 control and Catalpol‐treated organoids with or without TMZ. Arrows represent SOX2 + HOPX+ oRG cells. Scale bar, 20 μm (g) Quantification of SOX2 + HOPX+ oRG cells in oSVZ region on day 20 and 30 control and Catalpol‐treated organoids with or without TMZ. Data represent mean ± SEM. two‐way ANOVA, *p < 0.05, ****p < 0.0001; *n* = 3 per group. ns, not significant.

Next, we investigated how Catalpol promotes oRG formation in cerebral organoids. Previous reports have shown that oRGs arise from the asymmetric division of vRGs in the cortical germinal zones.^21^ Specifically, a vRG division angle within the vertical range (60–90°) indicates proliferation of vRGs, while angles within the oblique (30–60°) or horizontal (0–30°) range suggest differentiation into oRGs (Figure 2a).^8^, ^22^ Thus, we evaluated whether Catalpol enhances the generation of oRG by promoting the asymmetrical division of vRG. We examined the vRG cell division angle through immunostaining, focusing on asymmetrical divisions with oblique and horizontal orientations at the apical membrane of the VZ region in the Catalpol treated cerebral organoids. In particular, we used pericentrin and phosphorylated vimentin (pVIM) staining to identify mitotic centrosomes and mark cells in the M phase, allowing us to specifically identify mitotic vRG cells by their ventricular location in cerebral organoids.^23^ Analysis of mitotic spindle orientation in vRG cells showed a significant increase in cells undergoing oblique or horizontal divisions in Catalpol‐treated organoids, as indicated by the positioning of two centrosomes (Figure 2b). This shift in division orientation suggests that Catalpol promotes asymmetric divisions, potentially contributing to the generation and expansion of oRG cells in the organoids. We observed that vertical division significantly decreased from 55% to 29%, while oblique and horizontal divisions significantly increased from 45% to 70% in Catalpol‐treated organoids, suggesting that Catalpol induces the generation of oRG cells through non‐vertical divisions of vRG cells (Figure 2c). Furthermore, TMZ treatment that effectively depletes rapidly dividing cells, including NPCs, was conducted to verify the impact of Catalpol on oRG generation of cerebral organoids. We observed a remarkable reduction in pVim‐ and pH 3‐positive mitotic cells in the ventricular zone for both control and Catalpol‐treated organoids upon TMZ treatment (Figure 2d,e). Subsequently, after an additional 10 days of culture after TMZ treatment, we found that SOX2 + HOPX+ oRG cells were significantly restored from mitotic vRG cells in Catalpol‐treated organoids (Figure 2f,g). This restoration indicates that Catalpol exerts a stronger direct effect on promoting oRG generation than its indirect effect on vRG cells, supported by the significant increase in Ki67+, HOPX+ cells in the oSVZ (Figure S1c,d). Taken together, these findings indicate that Catalpol induces generation of oRG in organoids from mitotic vRG cells asymmetrically located in ventricular zone.

### 2.3 Catalpol enhances the generation of oRG by activation of STAT3 signaling

**FIGURE 3:**
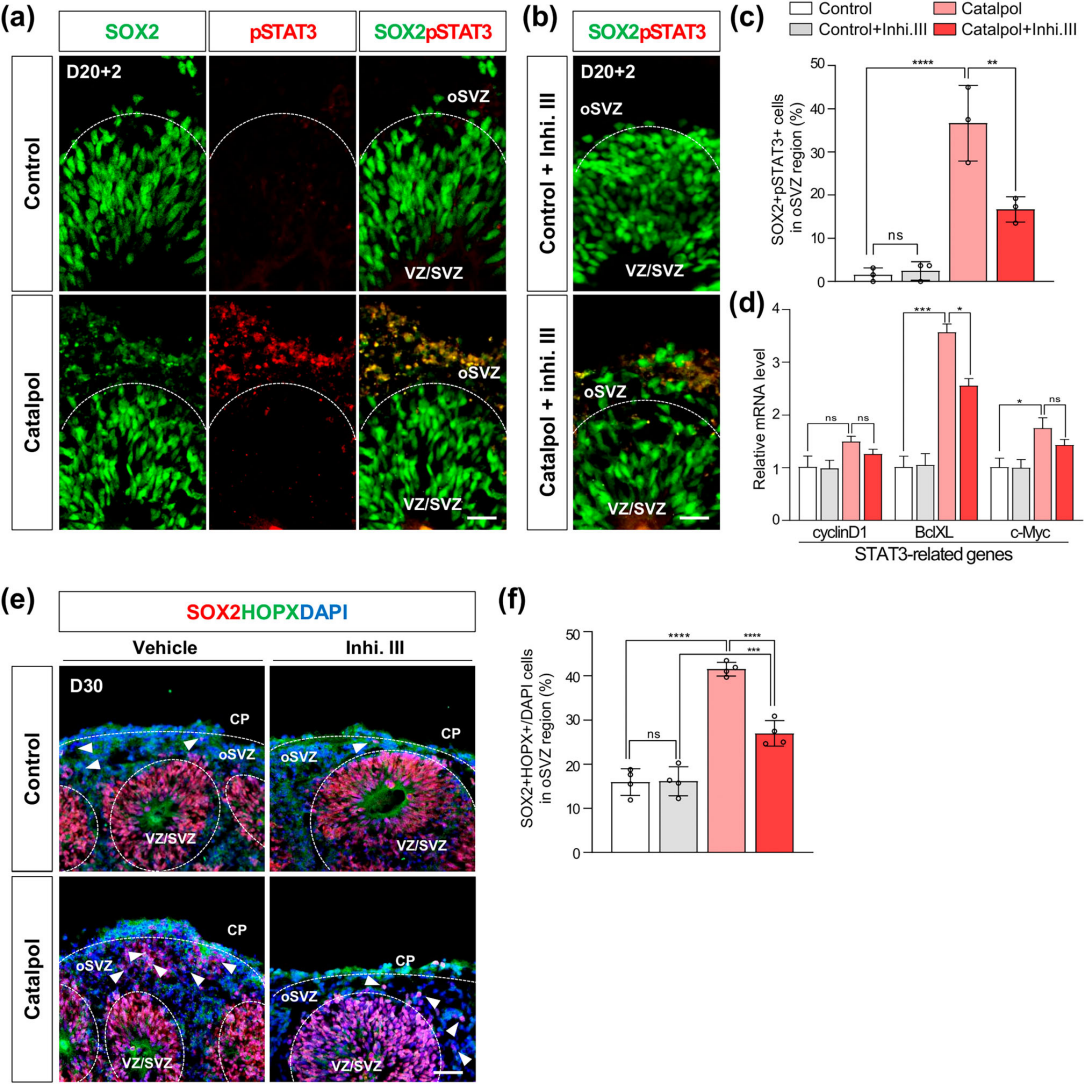
STAT3 activation by Catalpol promotes the generation of outer radial glia (oRG) in cerebral organoids. (a,b) Immunofluorescence staining for p‐Y705‐STAT3 (red) and SOX2 (green) in day 20 control and Catalpol‐treated organoids with or without STAT3 inhibitor III (Inhi. III). Scale bar, 20 μm. (c) Quantification of SOX2 + pSTAT3+ cells in oSVZ region. Data represent mean ± SEM. two‐way ANOVA, **p < 0.01, ****p < 0.0001; *n* = 3 per group. (d) Relative gene expression level of STAT3‐related genes (cyclinD1, BclXL, c‐Myc). Data represent mean ± SEM. two‐way ANOVA, *p < 0.05, ***p < 0.001; *n* = 3 per group. (e) Immunofluorescence staining for SOX2 (red), HOPX (green), and DAPI (blue) in day 30 control and Catalpol‐treated organoids with or without STAT3 inhibitor III (Inhi. III). Arrows represent SOX2 + HOPX+ oRG cells. Scale bar, 50 μm. (f) Quantification of SOX2 + HOPX+ oRG cells. Data represent mean ± SEM. Two‐way ANOVA, ***p < 0.001, ****p < 0.0001; *n* = 3 per group. ns, not significant.

Given the role of Catalpol in activating STAT3 signaling, we examined STAT3 phosphorylation levels in cerebral organoids. Interestingly, we observed that p‐STAT3 was specifically increased in the oSVZ region of Catalpol‐treated organoids compared to control organoids (Figure 3a). However, in the presence of a STAT3 phosphorylation inhibitor (inhibitor III), we observed a significant decrease in p‐STAT3 activation in the oSVZ region of Catalpol‐treated organoids, as confirmed by a reduction in the number of pSTAT3 + SOX2+ cells (Figure 3b,c). Consistent with these results, we further confirmed that the expression levels of STAT3‐related genes such as cyclinD1, BclXL, and c‐Myc, that increased by Catalpol treatment, were reduced by inhibitor III treatment (Figure 3d). Moreover, the number of SOX2 + HOPX+ oRG cells in the oSVZ region of Catalpol‐treated organoids was significantly decreased by inhibition of STAT3 activation (Figure 3e,f). Together, these results indicate that Catalpol enhances the generation of oRGs in cerebral organoids through the activation of STAT3 signaling.

### 2.4 Molecular mechanism of Catalpol mediated oRG generation in cerebral organoids

**FIGURE 4:**
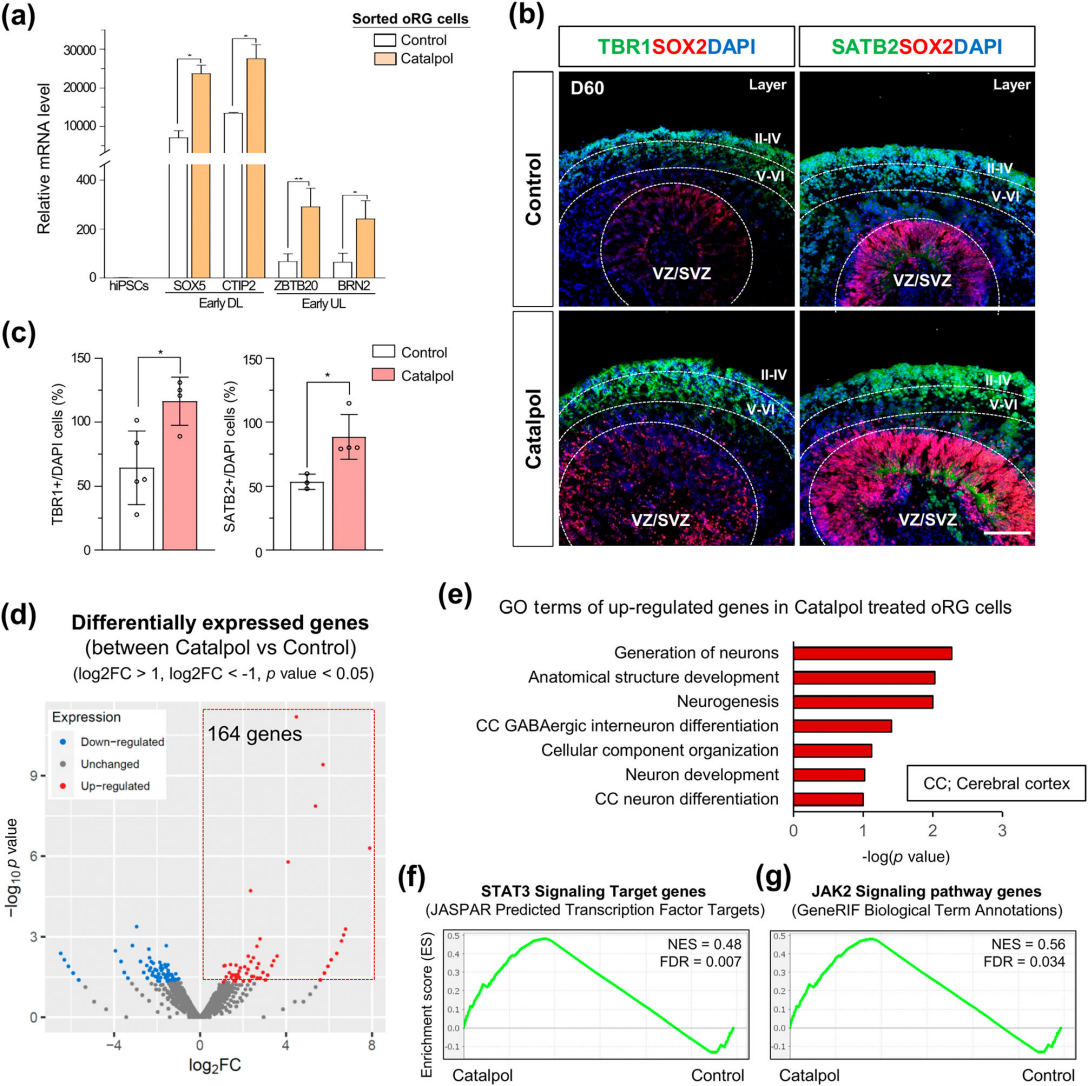
Catalpol enhances the neurogenic capacity of cerebral organoids during cortex development. (a) Relative gene expression level of early deeper layer (SOX5, CTIP2) and early layer (ZBTB20, BRN2) in sorted outer radial glia (oRG) cells from day 30 control and Catalpol‐treated organoids. Data represent mean ± SEM. Two‐tailed Student's t‐test, *p < 0.05, **p < 0.01; *n* = 3 per group. (b) Immunofluorescence staining for TBR1 (green), SATB2 (green), SOX2 (red), and DAPI (blue) in day 60 control and Catalpol‐treated organoids. The dotted lines highlight the regions of the VZ/SVZ, and layers (V–VI, II–IV) of cortex regions. Scale bar, 50 μm. (c) Quantification of TBR1+ cells (V–VI layer) and SATB2+ cells (II–IV layer) in day 60 control and Catalpol‐treated organoids. Data represent mean ± SEM. two‐tailed Student's t‐test, *p < 0.05; *n* = 4 per group. (d) Volcano plot for Catalpol‐treated versus control differentially expressed genes (DEGs) in human cerebral organoids (log2FC >1, log2FC < −1, *p* value <0.05). (e) Bar graph showing GO categories from upregulated genes in Catalpol treated organoid. (f) The GSEA plot indicates a positive correlation between STAT3 signaling pathway target genes and differentially expressed genes in Catalpol‐treated condition. (NES = 0.48, FDR‐q value = 0.007). (g) The GSEA plot indicates a positive correlation between JAK2 signaling pathway target genes and differentially expressed genes in Catalpol‐treated condition. (NES = 0.56, FDR‐q value = 0.034).

To further investigate the molecular mechanism underlying oRG generation in Catalpol‐treated organoids, we first sorted LIFR+ oRG cells from the Catalpol‐treated organoids using FACS (Figure S3b). FACS gating strategies ensured the isolation of LIFR+ cells by excluding debris (P1), identifying single cells (P2), and isolating LIFR+ populations while excluding IgG controls (P3) (Figure S3b). This approach allowed us to examine the development of oRG‐derived early and upper cortical layers in Catalpol‐treated organoids. In these sorted cells, we identified the expression of early deeper layer markers (SOX5, CTIP2) and early upper layer markers (ZBTB20, BRN2) and found a significant increase in early deeper and upper layer markers compared to control by the Catalpol treatment (Figure 4a). Additionally, at the late stage in cortical development shown in the cerebral organoids cultured for 60 days, we consistently observed significant increase in the TBR1+ and SATB2+ cells, deeper and upper layer markers, respectively by the Catalpol treatment (Figure 4b,c). Moreover, immunofluorescence analysis revealed that SOX2 + HOPX+LIFR+ oRG cells were prominently localized in the oSVZ region of day 30 Catalpol‐treated organoids (Figure S3a). To further evaluate the neural activity of organoids, micro‐electrode array (MEA) was performed,^24^ as evidenced by the generation of spontaneous neural activity in Catalpol‐treated organoids (Figure S4a). Together, these results indicate that the generation of oRG cells by Catalpol increases their capacity to differentiate into functional neurons with both deeper and upper layers during cortical development.

In addition, to explore the transcriptional properties induced by Catalpol treatment in the generation of human cerebral organoids with oRGs, we performed RNA sequencing on control and Catalpol‐treated cerebral organoids. Transcriptome analysis identified 327 differentially expressed genes (DEGs) (adjusted *p* < 0.05, |log2FC| < −1). Notably, a significant proportion of these DEGs were upregulated and 164 genes demonstrated robust expression changes, potentially indicating critical regulatory functions in the observed phenotype (Figure 4d). Moreover, we found that upregulated genes in Catalpol‐treated organoids indicated that neurogenic capacity signatures including generation of neurons, neurogenesis and cerebral cortex neuron differentiation (Figure 4e). Interestingly, we found that STAT3 signaling target genes were positively enriched in Catalpol‐treated condition by gene set enrichment analysis (GSEA) (Figure 4f). Additionally, we confirmed that target genes of the JAK2 signaling pathway, specifically activating and phosphorylating STAT3,^25^ were also activated by Catalpol treatment in human cerebral organoids (Figure 4g). These findings suggest that Catalpol influences neurogenic capacity through activation of JAK2/STAT3 signaling in the generation of human cerebral organoids with oRGs.

## 3 DISCUSSION

Our study provides compelling evidence that Catalpol, an iridoid glucoside from *Rehmannia glutinosa*, significantly enhances the generation of cerebral organoids enriched with oRG cells via activation of STAT3 signaling. Our findings show that Catalpol‐treated organoids have well‐defined oSVZ regions with increased thickness and a higher number of oRG cells, as shown by increased expression of oRG markers such as HOPX and FAM107A. Additionally, Catalpol influences the division angle of vRG cells, favoring non‐vertical divisions associated with oRG cells generation. Activation of STAT3 signaling is critical in this process, as inhibition of STAT3 phosphorylation reduces the oRG population in treated organoids. Furthermore, Catalpol enhances the neurogenic capacity of organoids, promoting differentiation into both early deeper and upper cortical layers. This was supported by increased expression of respective markers and RNA sequencing data indicating activation of STAT3‐related pathways, further supporting the role of Catalpol in augmenting neurogenic outcomes.

Generating cerebral organoids with consistent oRG populations and uniform structural features remains challenging due to the inherent variability in iPSC lines and differentiation processes.^9^, ^20^ This variability can lead to significant differences in organoid development, affecting their utility in modeling human brain development and diseases. Moreover, ensuring the proper maturation of these organoids into larger neural networks is also crucial for their application in disease modeling and regenerative medicine.^26^, ^27^ Thus, the use of Catalpol to promote the generation of cerebral organoids with oRG cells offers a potential solution to these challenges. By enhancing the formation of cerebral organoids with oRG cells and promoting more consistent organoid development, Catalpol can improve the reproducibility and scalability of brain organoid production. Moreover, the effects of Catalpol to activate STAT3 signaling suggest that pharmacological approaches can standardize and optimize the differentiation process. This can lead to more reliable models for studying neurodevelopment and neurological diseases, improving the robustness of experimental findings.

A key observation from our study is the shift in the mitotic spindle orientation of vRG cells toward oblique and horizontal angles upon Catalpol treatment. This shift suggests that Catalpol promotes oRG generation by favoring the non‐vertical, asymmetric division of vRG cells. The decrease in vertical divisions and the concurrent increase in oblique and horizontal divisions provide a mechanistic insight into how Catalpol influences oRG cell fate decisions. Moreover, Catalpol efficiently generates new oRG cells even after mitotic disruption by TMZ. This indicates that Catalpol may have a strong neurogenesis effects, promoting the differentiation of mitotic vRG cells in the ventricular zone.

Additionally, the role of STAT3 signaling in this process is further underscored by our results showing specific activation of phosphorylated STAT3 (p‐STAT3) in the oSVZ region of Catalpol‐treated organoids. The use of a STAT3 phosphorylation inhibitor significantly reduced the number of SOX2 + HOPX+ oRG cells, confirming that STAT3 activation is essential for the effects observed with Catalpol treatment. However, the number of oRG cells in the Catalpol‐treated organoids with STAT3 inhibitor treatment was still higher than in the control organoids, suggesting that additional signaling pathways may contribute to Catalpol‐induced oRG expansion. This indicates that, while STAT3 activation is crucial, other pathways, such as mTOR or ERK signaling, might also be involved in promoting oRG cell proliferation in response to Catalpol treatment.^28^, ^29^ The upregulation of genes associated with neurogenesis and cortical neuron differentiation, along with the enrichment of STAT3 and JAK2 signaling pathways, provides a molecular basis for the observed phenotypic changes. Previous studies demonstrated the importance of STAT3 in maintaining neural progenitor populations and promoting gliogenesis.^30^, ^31^ Thus, our results highlight the role of STAT3/JAK2 signaling in the neurogenic capacity of cerebral organoids with oRGs.

While Catalpol has been shown to promote neuronal differentiation, it is important to consider that the observed increase in mature neurons could be partly due to an expansion in neuroblasts originating from oRG cells. oRG cells are known to serve as progenitors that can give rise to neuroblasts, which then differentiate into neurons in both deep and upper cortical layers. This possibility consisted with other studies that demonstrated capacity of oRG cells to contribute to neurogenesis through the generation of neuroblasts.^5^, ^32^, ^33^ Consistently, we observed an increased expression of the neuroblast marker DCX, along with TBR2, which also marks cells with neuroblasts, clarifying that Catalpol‐induced increases in mature neurons are mediated by oRG‐derived neuroblasts.

In conclusion, this study demonstrates that Catalpol effectively promotes the generation of cerebral organoids with enhanced oRG populations and neurogenic potential through the activation of STAT3 signaling. Future studies should explore the long‐term effects of Catalpol treatment on cerebral organoid development and its potential applications in disease modeling and drug discovery. By enhancing the formation of oRG cells and promoting more consistent organoid development, we can improve the reproducibility and scalability of brain organoid production.

## 4 MATERIALS AND METHODS

### 4.1 Human iPSCs culture and generation of cerebral organoids

Human iPSCs cells were purchased from Coriell (GM23716) and maintained with StemMACS (Millipore) on Matrigel‐coated plates. Cells (9000 cells per well) were collected using versene (Gibco) and seeded into a 96‐well low‐attachment U‐bottom plate (Corning) for 3D cerebral organoid generation, according to the manufacturer's protocols (STEMCELL). In brief, organoids were initially cultured in Neurobasal medium (ThermoFisher) supplemented with 1% B27 (without vitamin A), 1% N2, and 1% GlutaMAX, along with dual SMAD inhibitors (SB431542 and LDN193189) to promote neuroectodermal induction until day 7. To support neuroepithelial bud formation, organoids were transferred to a medium containing DMEM/F12 and Neurobasal (1:1), supplemented with 0.5% N2, 1% GlutaMAX, 0.5% NEAA, 1% B27 (without vitamin A), 1% penicillin/streptomycin, 0.1 mM β‐mercaptoethanol, and 4 mg/mL insulin, and cultured under these conditions until day 10. From day 10 onward, organoids were cultured in differentiation medium, where the B27 supplement without vitamin A was replaced by B27 containing vitamin A, to support further maturation and differentiation. Organoids were maintained on a rotating orbital shaker with media changes every 2–3 days until day 30. Catalpol (10 μM; MedChemExpress) treatment was initiated on day 7 and maintained continuously in the culture medium until day 30. During this period, organoids were maintained in a rotating bioreactor, with media changes performed every 2–3 days.

### 4.2 Immunostaining of cerebral organoids

Organoids were washed with 1X PBS and fixed with 4% PFA at 4°C for overnight. Fixed organoids were washed with 1X PBST (0.1% Tween® 20 in PBS) and incubated with 30% sucrose until saturated. Organoids with embedded in optimal cutting temperature (OCT) compound (TissueTek) and sectioned on with a cryostat at 15 μm. For immunostaining, slides were washed with 1X PBST and fixed with 4% PFA for 15 min, and blocked with 3% BSA in 1X PBST for 1 h. The following primary antibodies were used: anti‐TBR1 (ab183032, Abcam), anti‐SOX2 (14‐9811‐82, Invitrogen), anti‐MAP2 (13‐1500, invitrogen), anti‐TBR2 (Ab23345, abcam), anti‐HOPX (FNab03970, FineTest), anti‐TNC (ab108930, Abcam), anti‐GFAP (sc‐33,673, Santa Cruz), anti‐Nestin (MA1‐100, Invitrogen), anti‐DCX (4604, cell signaling), anti‐NeuN (MAP377, Millipore), anti‐FOXG1 (ab18259, Abcam), anti‐PAX6 (42‐6000, Invitrogen), anti‐TUJ‐1 (T2200, Sigma), anti‐pericentrin (ab4448, Abcam), anti‐pVIM (phospho‐Vimentin; D076‐3 s (Ser55), MBL), anti‐pH 3 (9701, Cell Signaling), anti‐pSTAT3 (9145S, Cell Signaling), anti‐SATB2 (sc‐81,376, Santa Cruz). Primary antibodies diluted in 1% BSA in 1X PBST and incubated at 4°C for overnight. After washing, secondary antibodies (Invitrogen) were incubated for 1 h at room temperature. Finally, nuclei were labeled with 6‐diamidino‐2‐phenylindole (DAPI, Invitrogen) and sections were mounted in Fluoromount‐G mounting medium (VectorLabs) and images were captured using a confocal laser scanning microscope (ZEISS, LSM800).

### 4.3 Quantitative PCR

Total RNA were isolated from organoids using RNA mini pre kit (Philekorea). To synthesize complementary DNA from isolated RNA, the AccuPower RT‐PCR PreMix (Bioneer) was used according to the manufacturer's protocols. Quantitative RT‐PCR was performed using Platinum SYBR green qPCR SuperMix (Invitrogen) in a Rotor‐Gene Q real‐time PCR cycler (QIAGEN). Expression of all target genes was normalized against the expression of glyceraldehyde‐3‐phosphate dehydrogenase, GAPDH, in each organoid sample.

### 4.4 TMZ assay

To test the mitotic cells in organoids, 20 μM temozolomide (TMZ; MedchemExpress) was treated both day 10 and day 20 for 24 h. After that, the organoids were prepared for immunostaining with pVIM, pH 3 and SOX2. The organoids were cultured until the day of assay and prepared for immunostaining with SOX2 and HOPX.

### 4.5 STAT3 signaling

To examine the function of STAT3 signaling in oRG cells, Catalpol (10 μM) was treated for 48 h with or without or STAT3 inhibitor III (10 μM; sc‐203,282, Santa Cruz) on day 20. After that, the organoids were prepared for immunostaining with pSTAT3 and SOX2.

### 4.6 Organoids dissociation and FACS sorting

For sorting of oRG cells in cerebral organoids, organoids were dissociated into single cells using a papain dissociation kit (Worthington). The organoids were dissected into small pieces, incubated with papain and DNase I solution for 35–45 min, triturated and the cell suspension was filtered twice through 40‐μm filter to obtain a single‐cell suspension. The cells were centrifuged at 300 g for 5 min, and pellet was resuspended in 1X PBS containing 0.4% BSA and counted for viability (>90%). The cells were incubated with blocking buffer (2% FBS in 1X PBS) at 4°C for 30 min and centrifuged at 300 g for 5 min. The cell pellet was resuspended with FACS buffer containing LIFR‐conjugated antibody (FAB249P, R&D systems) for 1 h. After washing with 1X PBS for three times, cells were resuspended with FACS buffer (1% BSA and 0.01% sodium azide in 1X PBS).

### 4.7 Bulk RNA sequencing and analysis

For bulk RNA sequencing, total RNA from cerebral organoids was extracted on day 30. RNA integrity was assessed before library preparation using the TruSeq Stranded mRNA Sample Prep Kit according to the manufacturer's instructions (Illumina). The libraries were sequenced in a paired‐end fashion with 100 bp reads on the Illumina HiSeq 2500 platform. Alignment was carried out using STAR, while raw counts were generated utilizing bowtie2. Subsequently, variance stabilizing transformation normalization (vst) and differential expression analyses were conducted using DESeq2. For gene set enrichment analysis (GSEA), STAT3 pathway target gene sets were obtained via JASPAR Predicted Transcription Factor Targets. JAK2 pathway target gene sets were obtained via GeneRIF Biological Term Annotations.

### 4.8 MEA recording

The method for recording spontaneous neuronal activity in cerebral organoids was adapted from techniques described in the study by Lee et al., 2022.^24^ In brief, cerebral organoids treated with Catalpol were placed on a 60‐channel MEA plate (Axion BioSystems). Prior to recording, the organoids were allowed to acclimate in the MEA chamber, maintained at 37°C and 5% CO₂, for a minimum of 10 min. Spontaneous neuronal activity was recorded for 10 min using the Maestro system. Data, including neuronal spikes and network activity, were collected and analyzed with AxIS software, following similar procedures to those described in the referenced study. Selected sections of the recordings were magnified for detailed analysis of specific spiking events.

### 4.9 Statistical analysis

All data are presented as the mean ± SEM of three independent biological replicates (*n* = 3 per group), unless otherwise specified. Each biological replicate was performed using independently generated organoids to ensure the reproducibility and robustness of the findings. Values of ‘*n*’ indicate the number of independent experiments performed or the number of individual experiments. Statistical analyses were performed with GraphPad Prism. The *p*‐values were calculated by Student's t‐test and two‐way ANOVA tests followed by Tukey–Kramer multiple comparisons test. All of the statistical details for each experiment have been documented in the individual figure legends.

## 5 CONCLUSIONS

This study demonstrates that Catalpol, derived from *Rehmannia glutinosa*, significantly enhances the generation of cerebral organoids with expanded oRG populations. Catalpol‐treated organoids showed increased thickness and higher numbers of oRG cells in well‐defined oSVZ. We found that oRG cells were promoted by non‐vertical divisions of mitotic vRG cells. This effect is mediated through the activation of the STAT3 signaling pathway. These findings suggest that Catalpol improves the reproducibility and neurogenic potential of cerebral organoids, providing a valuable tool for more accurate modeling of brain development and neurodevelopmental disorders.

## AUTHOR CONTRIBUTIONS

Yoo‐Jung Lee performed the experiments. Byounggook Cho and Daeyeol Kwon performed the data analysis. Yoo‐Jung Lee and Jongpil Kim designed the study and Yoo‐Jung Lee, Yunkyung Kim, Saemin An, Soi Kang and Jongpil Kim contributed to writing the manuscript.

## CONFLICT OF INTEREST STATEMENT

The authors declare no conflict of interest.

## Supporting information


**Data S1.** Supporting Information.

## Data Availability

The bulk RNA‐seq datasets generated in this study have been deposited in the SRA accession numbers (SRR2967964, SRR29672965).
