# Supplementary material for: Catalpol promotes the generation of cerebral organoids with oRGs through activation of STAT3 signaling
Source: Bioeng Transl Med. 2024 Dec 29;10(3):e10746. doi: 10.1002/btm2.10746 (PMC12079450; doi:10.1002/btm2.10746)
Supplement: Supplementary file 1 — Data S1. Supporting Information. [file BTM2-10-e10746-s001.docx]

**Supporting information**


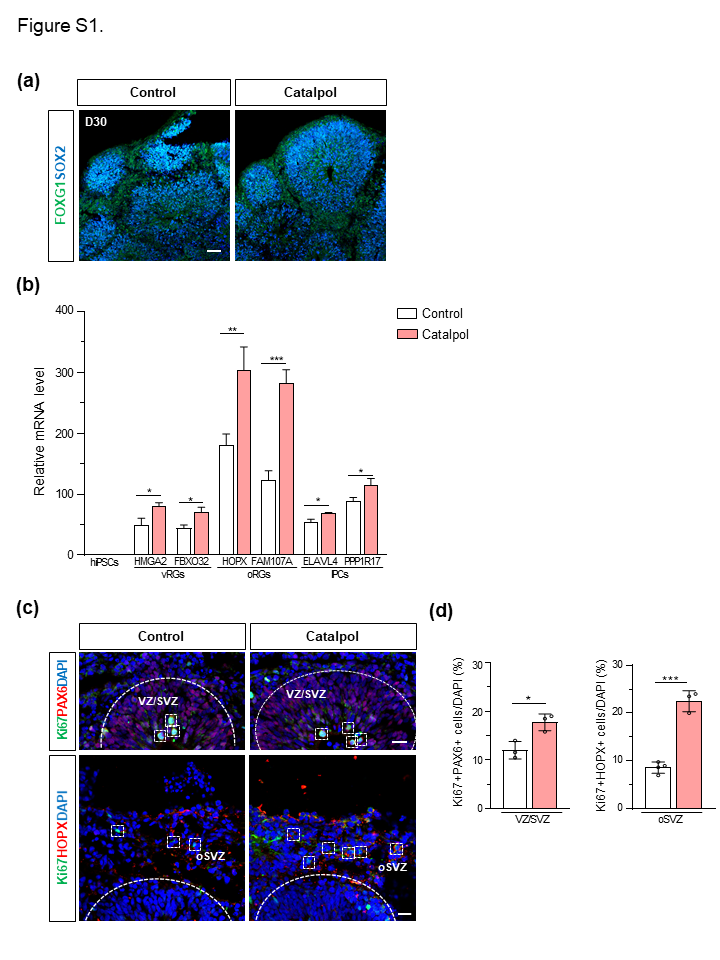


**FIGURE S1. Forebrain identity and the gene expression of cortical developmental markers in cerebral organoids**

(a) Immunofluorescence staining for FOXG1 (green) and SOX2 (blue) in day 30 control and Catalpol-treated organoids. Scale bar, 50 µm. (b) Relative gene expression level of vRG (HMGA2, FBZXO32), oRG (HOPX, FAM107A), and IPC (ELAVL4, PPP1R17) markers in day 30 control and Catalpol-treated organoids. Data represent mean ± SEM. two-tailed Student’s t-test, *p < 0.05, **p < 0.01, and ***p < 0.001; n = 3 per group. (c) Immunofluorescence staining for Ki67 (green), PAX6+ (red), HOPX (red), and DAPI (blue) in day 30 control and Catalpol-treated organoids. Scale bar, 20 µm (d) Quantification of Ki67+PAX6+ cells in the VZ/SVZ and Ki67+HOPZ+ in the oSVZ regions on day 30 control and Catalpol-treated organoids. Data represent mean ± SEM. two-way ANOVA, *P < 0.05, ***P < 0.001; n = 3 per group.


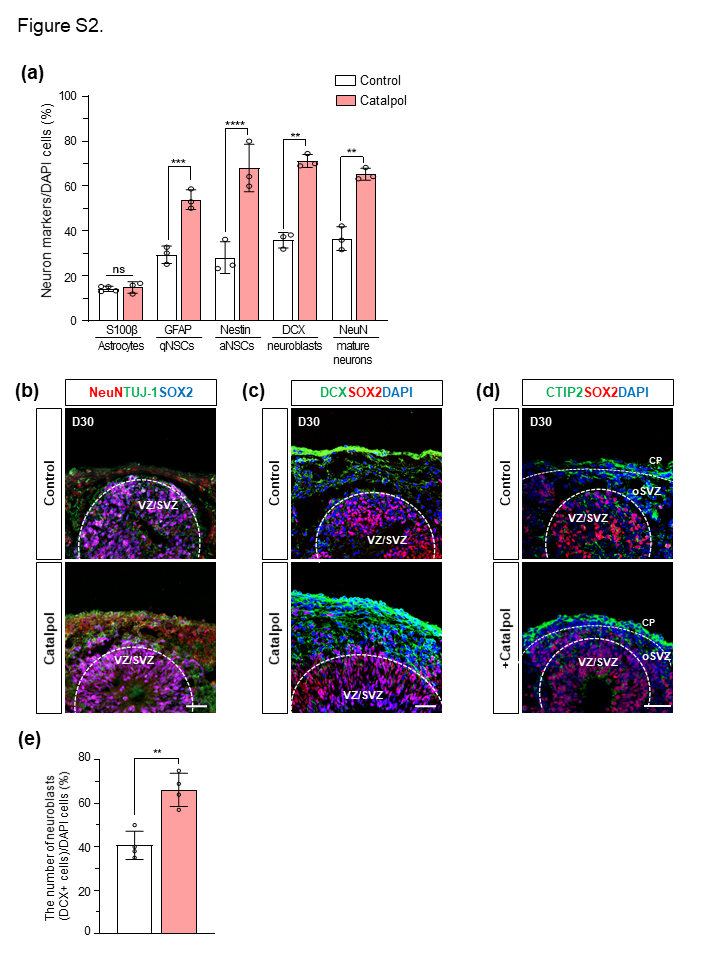


**FIGURE S2. Neurogenic capacity in cerebral organoids**

(a) Quantification of astrocytes (S100β+), qNSCs (GFAP+), aNSCs (Nestin+), neuroblasts (DCX+), and mature neurons (NeuN+) markers in day 30 control and Catalpol-treated organoids. Data represent mean ± SEM. two-way ANOVA, *P < 0.05, **P < 0.01, ***P < 0.001, ****P < 0.0001; n = 3 per group. Not significant, ns. Immunofluorescence staining for (b) NeuN (red), TUJ-1 (green), and SOX2 (blue) (c) DCX (green), SOX2 (red), and DAPI (blue) (d) CTIP2 (green), SOX2 (red), and DAPI (blue) in day 30 control and Catalpol-treated organoids. Scale bar, 50 µm. (e) The number of neuroblast marker, DCX-positive cells, in day 30 control and Catalpol-treated organoids. **p < 0.01; n = 3 per group.


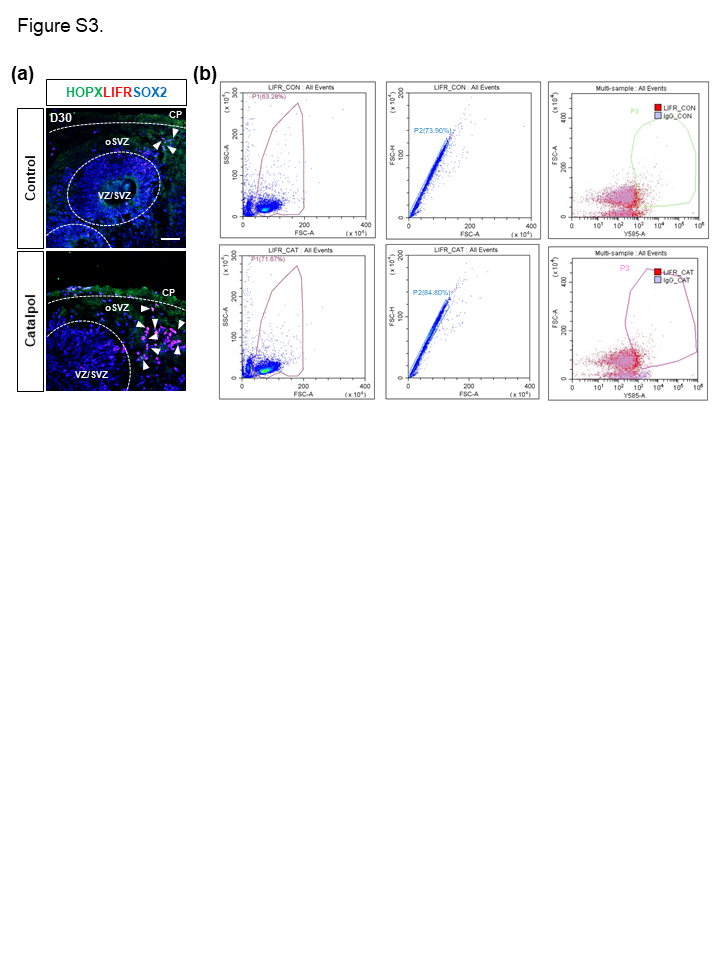


**FIGURE S3. LIFR expression and FACS isolation strategy**

(a) Immunofluorescence for HOPX (green), LIFR (red), and SOX2 (blue) in day 30 control and Catalpol-treated organoids. Arrows represent SOX2+HOPX+LIFR+ oRG cells. The dotted lines highlight the regions of the VZ/SVZ, oSVZ, and CP. Scale bar, 50 µm. (b) FACS isolation strategy for LIFR+ cells. Cells were gated from debris (P1) and single cells were then distinguished (P2) and LIFR+ cells, excluding the IgG control population, were subsequently isolated (P3).

**FIGURE S4. Electrophysiological analysis in Catalpol-treated organoids**


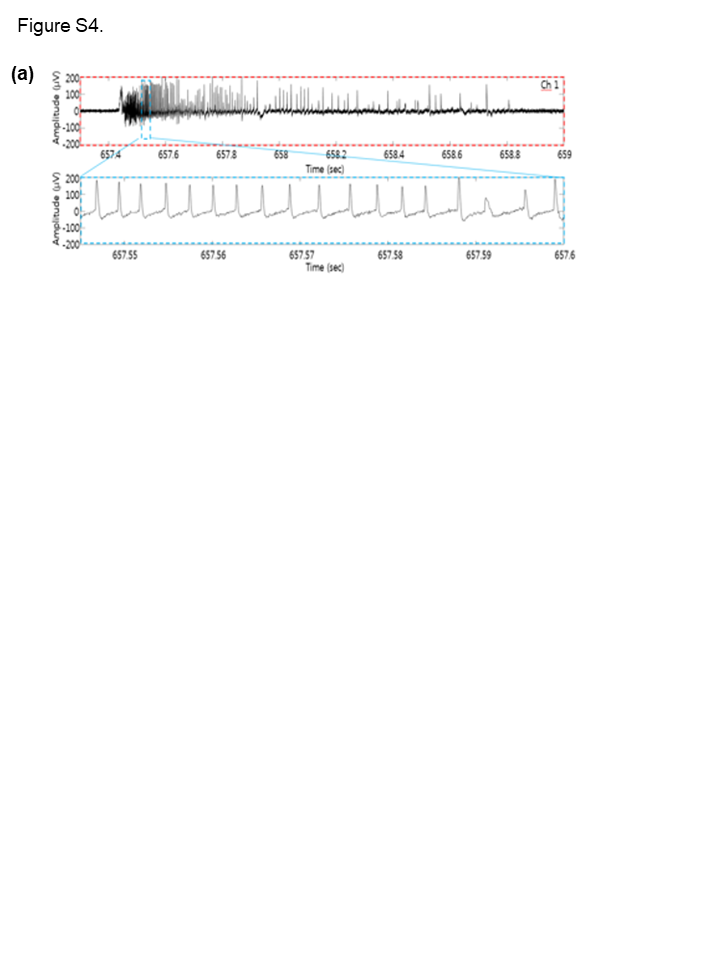


(a) The upper panel shows the overall spontaneous electrical activity recorded via micro-electrode array (MEA) over a 2-second time window. The lower panel provides an expanded view of the neuronal spikes, highlighting rhythmic spiking activity.
